# Book Reviews

**Published:** 1898-07

**Authors:** 


### Book Reviews.

A Manual of Hygiene and Sanitation. By Seneca Egbert, A.M., M.D., Professor of Hygiene and Dean of the Medico-Chirurgical College of Philadelphia; Professor of Anatomy, Physiology and Hygiene in Temple College; member of the Academy of Natural Sciences of Philadelphia, etc., etc. Illustrated with sixty-three engravings. Lea Brothers & Co., Philadelphia and New York. 1898.

The growing importance of hygiene and sanitation in relation to personal and public good health is evidenced by the number of treatises appearing upon these subjects. Constant activity in this direction, the addition of new facts are further factors in explaining the abundance of the literature on hygienic and sanitary matters. Despite this, there is always a place for a good manual containing up-to-date information and brief enough not to weary the reader. It seems to us that in this volume of less than 400 pages these objects have been attained.

The author's experience as a teacher leads him to appreciate the wants of the students of medicine as well as the general reader, and enables him to present the salient points of his subject without wasting space over that which is not absolutely required.

The subect is considered under the following heads: "Bacteriology," showing the importance of the germ theory in relato hygiene and sanitation and containing the essentials of bacteriological study; "The Atmosphere," showing the composition of the air and the agents of contamination and the effects of such impurities; "Ventilation and Healing;" a valuable chapter on water; also one on food. These are followed by chapters on personal and school hygiene; disinfection and quinine: the removal and disposal of sewerage; vital statistics, and one containing technics of examining air, water and food.

The clearness and succinctness of the language, the wealth of explanatory illustrations and the brevity without important omission recommend the work as a text-book, and as such we predict for it a notable popularity.

---

A Manual of General Pathology for Students and Practitioners. By Walter Sydney Lazarus Barlow, B.A., B.C., M.D., M.R.C.P. Late Demonstrator of Pathology and Examiner in Sanitary Science in the University. Philadelphia: P. Blakiston, Son & Co. 1898.

The author's endeavor is to supply a deficiency which exists in the literature of General or Experimental Pathology. His scholarly production supplies the need.

The work is concerned with the basic principles of medicine gained from experiment and observation and applied to the explanation of the varied processes of disease. To those whose education in medicine was before the rise of pathology and microscopy, this book is especially to be recommended. It will throw light upon subjects obscure to those who are forced to view them solely from the empirical, clinical standpoint. To the modern student such knowledge is essential. The volume is a masterly exposition of the subject, and will doubtless do much good in instructing many who are inclined to seek rather for drugs than causes of diseases.

---

A TEXT-BOOK OF MEDICAL JURISPRUDENCE AND TOXICOLOGY. By John J. Reese, M.D., late Professor of Medical Jurisprudence and Toxicology in the University of Pennsylvania; late President of the Medical Jurisprudence Society of Philadelphia. Fifth edition, revised by Henry Leffman, A.M., M.D., Ph. D., Professor of Chemistry and Toxicology in the Woman's Medical College of Pennsylvania, Pathological Chemist to the Jefferson College Hospital, Philadelphia. Philadelphia: P. Blakiston, Son & Co. 1898.

The popularity attained by previous editions of this well-known work insures a cordial reception for the present revision. The conservative nature of medical jurisprudence being so closely allied to the law, precludes the addition to the subject of general facts hitherto unknown. The work of revision has consisted in reports of new and difficult cases not included in the former editions, as well as in noting the advances made possible by the application of the X-Rays and of the laws relating to the medical profession, medical education and sanitation, which are constantly increasing.

The toxicologic portion has been carefully revised by the editor, whose personal work in this connection is well and favorably known.

---

TWENTIETH CENTURY PRACTICE OF MEDICINE. Vol. XIV. Infectious Diseases. Wm. Wood & Co., New York.

The contributors to this volume and their contributions are as follows:

Dillon Brown, M.D., New York, "Chicken-pox."

David Bruce, M.B., C.M., Pietermaritzburg, South Africa, "Malta Fever."

Sir Joseph Fayrer, Bart., M.D., LL.D., F.R.S., F.R.C.P., F.R C.S., London, "Dengue."

F. Forchheimer, M.D., Cincinnati, O., "Scarlet Fever."

A. Jacobi, M.D., New York, "Cholera Infantum," an especially valuable contribution.

A. Nelter, M.D., Paris, "Military Fever."

N. B. Norton and Joseph O'Dwyer, M.D., New York, "Whooping Cough."

Theodor Rumpf, M.D., Hamburg, "Asiatic Cholera."

A. A. DeAzevedo Sodre, Rio de Janeiro, "Beri-beri."

Dawson Williams, M.D., M.R.C.P., London, "Measles."

---

BRIEF ESSAYS ON ORTHOPEDIC SURGERY. By Newton U. Shaffer, M.D. New York: D. Appleton & Co. 1898.

These essays are a collection of papers by the author, which have appeared from time to time in the medical press. Their titles are as follows: "Present Status of Orthopedic Surgery," "What is Orthopedic Surgery?" "On the Definition and Scope of Orthopedic Surgery," "The Relation of Orthopedic to General Surgery," "Present Needs of Orthopedic Surgery," "Is Orthopedic Surgery to Become an Obsolete Specialty?" with remarks on specialism. The essays are all interesting and show that the author has his heart in his work, and has proven an able defender of it against its critics.

---

### BOOKS RECEIVED.

Physical Diagnosis—Tyson. P. Blakiston, Son & Co.

Materia Medica for Nurses—Groff. *Ib.*

Exercises in Pathology—Whitacre. *Ib.*

Quiz Compends. Diseases of the Skin—Schamberg. *Ib.*

Diseases of Children—Taylor & Wells. *Ib.*

Conservative Gynecology—Massey. F. A. Davis Co.

